# A Critical Review of Consumer Wearables, Mobile Applications, and Equipment for Providing Biofeedback, Monitoring Stress, and Sleep in Physically Active Populations

**DOI:** 10.3389/fphys.2018.00743

**Published:** 2018-06-28

**Authors:** Jonathan M. Peake, Graham Kerr, John P. Sullivan

**Affiliations:** ^1^Tissue Repair and Translational Physiology Research Program, School of Biomedical Sciences and Institute of Health and Biomedical Innovation, Queensland University of Technology, Brisbane, QLD, Australia; ^2^Sport Performance Innovation and Knowledge Excellence, Queensland Academy of Sport, Brisbane, QLD, Australia; ^3^Movement Neuroscience and Injury Prevention Program, Institute of Health and Biomedical Innovation, Queensland University of Technology, Brisbane, QLD, Australia; ^4^Clinical and Sports Consulting Services, Providence, RI, United States

**Keywords:** health, performance, stress, emotion, sleep, cognitive function, concussion

## Abstract

The commercial market for technologies to monitor and improve personal health and sports performance is ever expanding. A wide range of smart watches, bands, garments, and patches with embedded sensors, small portable devices and mobile applications now exist to record and provide users with feedback on many different physical performance variables. These variables include cardiorespiratory function, movement patterns, sweat analysis, tissue oxygenation, sleep, emotional state, and changes in cognitive function following concussion. In this review, we have summarized the features and evaluated the characteristics of a cross-section of technologies for health and sports performance according to what the technology is claimed to do, whether it has been validated and is reliable, and if it is suitable for general consumer use. Consumers who are choosing new technology should consider whether it (1) produces desirable (or non-desirable) outcomes, (2) has been developed based on real-world need, and (3) has been tested and proven effective in applied studies in different settings. Among the technologies included in this review, more than half have not been validated through independent research. Only 5% of the technologies have been formally validated. Around 10% of technologies have been developed for and used in research. The value of such technologies for consumer use is debatable, however, because they may require extra time to set up and interpret the data they produce. Looking to the future, the rapidly expanding market of health and sports performance technology has much to offer consumers. To create a competitive advantage, companies producing health and performance technologies should consult with consumers to identify real-world need, and invest in research to prove the effectiveness of their products. To get the best value, consumers should carefully select such products, not only based on their personal needs, but also according to the strength of supporting evidence and effectiveness of the products.

## Introduction

The number and availability of consumer technologies for evaluating physical and psychological health, training emotional awareness, monitoring sleep quality, and assessing cognitive function has increased dramatically in recent years. This technology is at various stages of development: some has been independently tested to determine its reliability and validity, whereas other technology has not been properly tested. Consumer technology is moving beyond basic measurement and telemetry of standard vital signs, and predictive algorithms based on static population-based information. Health and performance technology is now moving toward miniaturized sensors, integrated computing, and artificial intelligence. In this way, technology is becoming “smarter,” more personalized with the possibility of providing real-time feedback to users (Sawka and Friedl, 2018). Technology development has typically been driven by bioengineers. However, effective validation of technology for the “real world” and development of effective methods for processing data requires collaboration with mathematicians and physiologists (Sawka and Friedl, 2018).

**Figure 1:**
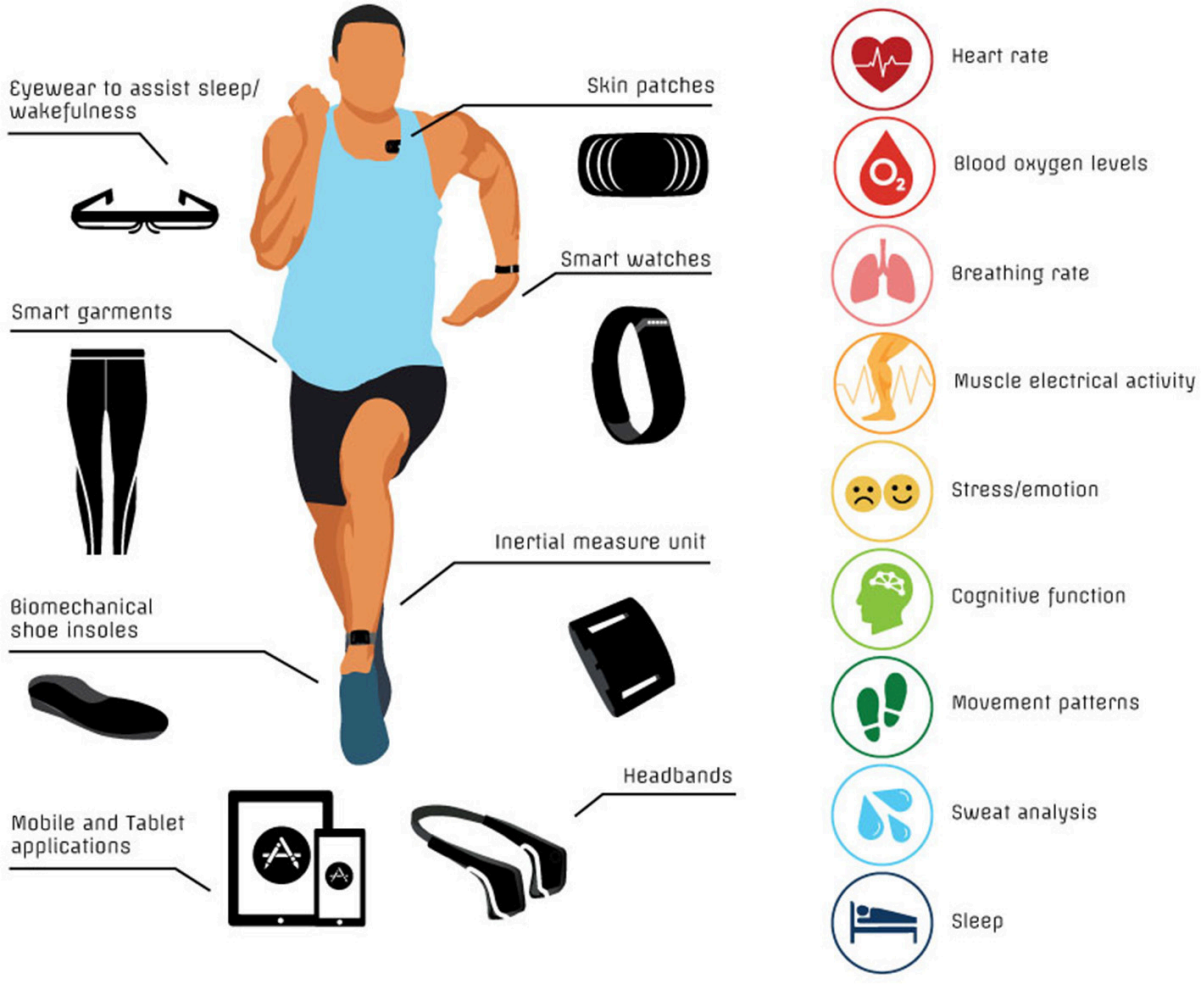
Summary of current technologies for monitoring health/performance and targeted physical measurements.

Although there is some overlap between certain technologies, there are also some differences, strengths and weaknesses between related technologies. Various academic reviews have summarized existing technologies (Duking et al., 2016; Halson et al., 2016; Piwek et al., 2016; Baron et al., 2017). However, the number and diversity of portable devices, wearable sensors and mobile applications is ever increasing and evolving. For this reason, regular technology updates are warranted. In this review, we describe and evaluate emerging technologies that may be of potential benefit for dedicated athletes, so-called “weekend warriors,” and others with a general interest in tracking their own health. To undertake this task, we compiled a list of known technologies for monitoring physiology, performance and health, including concussion. Devices for inclusion in the review were identified by searching the internet and databases of scientific literature (e.g., PubMed) using key terms such as “technology,” “hydration,” “sweat analysis,” “heart rate,” “respiration,” “biofeedback,” “respiration,” “muscle oxygenation,” “sleep,” “cognitive function,” and “concussion.” We examined the websites for commercial technologies for links to research, and where applicable, we sourced published research literature. We broadly divided the technologies into the following categories (Figure 1):

- devices for monitoring hydration status and metabolism
- devices, garments, and mobile applications for monitoring physical and psychological stress
- wearable devices that provide physical biofeedback (e.g., muscle stimulation, haptic feedback)
- devices that provide cognitive feedback and training
- devices and applications for monitoring and promoting sleep
- devices and applications for evaluating concussion.

Our review investigates the key issues of: (a) what the technology is *claimed* to do; (b) has the technology been independently validated against some accepted standard(s); (c) is the technology reliable and is any calibration needed, and (d) is it commercially available or still under development. Based on this information we have evaluated a range of technologies and provided some unbiased critical comments. The list of products in this review is not exhaustive; it is intended to provide a cross-sectional summary of what is available in different technology categories.

## Devices for monitoring hydration status and metabolism

Several wearable and portable hardware devices have been developed to assess hydration status and metabolism, as described below and in Table 1. Very few of the devices have been independently validated to determine their accuracy and reliability. The Moxy device measures oxygen saturation levels in skeletal muscle. The PortaMon device measures oxy-, deoxy-, and total hemoglobin in skeletal muscle. These devices are based on principles of near infrared spectroscopy. The PortaMon device has been validated against phosphorus magnetic resonance spectroscopy (^31^P-MRS) (Ryan et al., 2013). A similar device (Oxymon) produced by the same company has been proven to produce reliable and reproducible measurements of muscle oxygen consumption both at rest (coefficient of variation 2.4%) and after exercise (coefficient of variation 10%) (Ryan et al., 2012). Another study using the Oxymon device to measure resting cerebral oxygenation reported good reliability in the short term (coefficient of variation 12.5%) and long term (coefficient of variation 15%) (Claassen et al., 2006). The main limitation of these devices is that some expertise is required to interpret the data that they produce. Also, although these devices are based on the same scientific principles, they do vary in terms of the data that they produce (McManus et al., 2018).

**Table 1:** Devices for monitoring hydration status and metabolism.

**Product category**	**Product name**	**Technical characteristics**	**Validated**	**Reliability testing**	**Calibration required**	**Development company/organization**	**Commercially available**
Smart watch	Hydra Alert HRM Hydration Monitor	Monitors hydration status. Sensors for detecting temperature and humidity. MAX MET (VO_2max_) calibration. Heat index calculator. Standard heart rate monitor and interval timer/countdown.	No	No	Not stated	Acumen™	Yes
Smart watch	Halo Edge	Monitors hydration status, activity levels, and environmental conditions.	No	No	Not stated	Halo Wearables	Yes
Strap/band/patch	Humon Hex	Strap with sensor paired to wristwatch and mobile application to measure muscle oxygenation levels.	No	No	No	Dynometrics Inc	Available mid 2017
Strap/band/patch	Nobo B60	Strap with sensor monitor hydration status.	No	No	Not stated	Nobo Inc.	In development
Strap/band/patch	ECHO™ Smart Patch	Wearable device to measure hydration, sodium, glucose, metabolites, various molecules, and proteins.	No	No	Not stated	Kenzen	In development
Strap/band/patch	BSX Insight	Sleeve with near infrared sensors to detect muscle oxygenation and lactate levels. Useful for determining lactate thresholds non-invasively. Connectable to ANT+ fitness tracking watches, mobile application, and computers.	Yes	Yes	No	BSX Athletics	Yes
Wearable device	PortaMon	Measures oxy- deoxy- and total hemoglobin, blood volume and blood flow, as well as tissue saturation in muscle tissue using near infrared spectroscopy. Bluetooth (150 m) or on-board data collection.	Independent published research	Yes	Yes	Artinis Medical Systems	Yes
Wearable device	Moxy	Uses near infrared spectroscopy to measure muscle oxygenation levels in muscle tissue. Lightweight (40 g) and water resistant. On-board data collection and wireless data transmission.	Independent published research	No	Yes	Fortiori Design	Yes
Non-wearable device	Breezing	Device linked to mobile application to measure respiratory quotient in exhaled breath as a measure of the balance of carbohydrate and fat metabolism. Measures and records history of energy expenditure.	No	No	Not stated	Breezing	Yes
Non-wearable device	The LEVL Device	Device linked to mobile application to measure the acetone content of exhaled breath as a measure of fat metabolism.	No	No	Not stated	Medamonitor LLC	Yes

*Device is considered the gold standard in its class i.e., no comparison with other technology is possible*.

The BSX Insight wearable sleeve has been tested independently (Borges and Driller, 2016). Compared with blood lactate measurements during a graded exercise test, this device has high to very high agreement (intraclass correlation coefficient >0.80). It also has very good reliability (intraclass correlation coefficient 0.97; coefficient of variation 1.2%) (Borges and Driller, 2016). This device likely offers some useful features for monitoring muscle oxygenation and lactate non-invasively during exercise. However, one limitation is that the sleeve that houses the device is currently designed only for placement on the calf, and may therefore not be usable for measuring muscle oxygenation in other muscle groups. The Humon Hex is a similar device for monitoring muscle oxygenation that is touted for its benefits in guiding warm-ups, monitoring exercise thresholds and recovery. For these devices, it is unclear how reference limits are set, or established for such functions.

Other non-wearable devices for monitoring metabolism, such as Breezing and the LEVL device, only provide static measurements, and are therefore unlikely to be useful for measuring metabolism in athletes while they exercise. Sweat pads/patches have been developed at academic institutions for measuring skin temperature, pH, electrolytes, glucose, and cortisol (Gao et al., 2016; Koh et al., 2016; Kinnamon et al., 2017). These devices have potential applications for measuring heat stress, dehydration and metabolism in athletes, soldiers, firefighters, and industrial laborers who exercise or work in hot environments. Although these products are not yet commercially available, they likely offer greater validity than existing commercial devices because they have passed through the rigorous academic peer review process for publication. Sweat may be used for more detailed metabolomic profiling, but there are many technical and practical issues to consider before this mode of bioanalysis can be adopted routinely (Hussain et al., 2017).

## Technologies for monitoring training loads, movement patterns, and injury risks

A wide range of small attachable devices, garments, shoe insoles, equipment, and mobile applications have been developed to monitor biomechanical variables and training loads (Table 2). Among biomechanical sensors, many are based around accelerometer and gyroscope technology. Some of the devices that attach to the body provide basic information about body position, movement velocity, jump height, force, power, work, and rotational movement. This data can be used by biomechanists and ergonomists to evaluate movement patterns, assess musculoskeletal fatigue profiles, identify potential risk factors for injury and adjust techniques while walking, running, jumping, throwing, and lifting. Thus, these devices have application in sporting, military and occupational settings.

**Table 2:** Devices and garments for monitoring training loads, movement patterns, and injury risks.

**Product category**	**Product name**	**Technical characteristics**	**Validated**	**Reliability testing**	**Calibration required**	**Development company/organization**	**Commercially available**
Wearable device	mPower	Pod containing electrodes placed on the skin (with strap or adhesive strips) to record surface EMG signals. Can be used to determine activation of different types of muscle fibers, muscle fatigue, timing of muscle activation relative to movement. Derived metrics include activation power, activation volume, active power balance, fatigue index. Connected to mobile application.	No	No	Uncertain	Fibrux Oy	Yes
Wearable device	Zephyr™	Sensor connected to a strap around the chest or imbedded within a singlet. Measures biomechanical data including posture, physical activity, peak acceleration, impact on the body, jump height and flight time, explosiveness, peak force, GPS speed, distance and elevation. Integrates data to provide a summary of physiological load/intensity, mechanical load, training load/intensity.	No	No	Uncertain	Medtronic	Yes
Wearable device	KuaiFit	Headphones that measure heart rate, speed, steps, distance, cycling cadence, swimming laps and strokes, calories. Audible training plans. Connected by Bluetooth and ANT+ to wristwatches, bike computers, mobile devices, gym equipment.	No	No	Not stated	KuaiFit	Not at present
Wearable device	Biostrap	Shoe clip with a three-axis accelerometer and gyroscope. Recognizes different exercise modes and quantifies the number of repetitions, exercise duration, form and consistency.	No	No	No	Biostrap USA, LLC	Yes
Wearable device	I Measure U	Clip with inertial sensor with three-axis accelerometer, gyroscope, and compass. Measures jump height, velocity, power, peak force, rate of force development, flight time, vertical displacement, number of steps, velocity, and number of barbell movements.	No	No	Not stated	I Measure U Ltd	Yes
Wearable device	PUSH	Accelerometer and gyroscope attached to a strap to record velocity, power, and total work.	No	No	Not stated	PUSH Inc	Yes
Wearable device	Lumo Run	Clip that attaches to shorts; clip contains 9-axis inertial measurement unit (IMU), accelerometer, gyroscope, magnetometer, and barometer. Provides data on cadence, braking, bounce, pelvic rotation, pelvic drop. Connected to mobile application.	No	No	Not stated	Lumo BodyTech	Yes
Garments	DynaFeed	Smart garment combining advanced biosensor technology with an ultra-thin conductive carbon nanotubes polymer film. Monitors heart rate, workout effort, provides real-time guidance to improve efficiency and avoid injuries.	No	No	Uncertain	Far Eastern New Century Corporation	No
Garments	Sensoria garments	Upper body garments that monitor heart rate; socks that monitor distance, cadence, foot landing, foot contact, pace. Connected to a mobile application that provides dashboard tracking and coaching.	No	No	Uncertain	Sensoria	Yes
Wearable and non-wearable devices	VERT	A range of devices for measuring vertical jump height, number of jumps, average height, jump rate, power, movement intensity, and asymmetry.	No	No	Not stated	VERT	Yes
Garments	Athos	Garments with embedded EMG sensors to measure muscle activity, muscle effort, and balance. Upper body garment measures heart rate Connected to mobile application.	No		Not stated	Mad Apparel Inc.	Yes
Wearable device	Mettis Trainer	Biomechanical shoe insoles containing force and pressure sensors; measure cadence, distance travelled, gait, weight distribution of foot landing, heel-to-toe-strike, impact force, contact time. Provide real-time audio feedback. Connected to mobile application.	No		“Self-calibrating”	Mettis Trainer	Yes
Wearable device	Arion	Biomechanical shoe insoles connected to a footpod, wristband, and mobile application. Record foot position, cadence, stride length.	No		Uncertain	ATO Gear	Not at present
Mobile application	Kinduct	Collects, processes and stores large amounts of data on athletes. Data analytics and visualization tools for identifying areas of strength, opportunities for improvement and potential risks for injury. Tracking, notification and communication tools for personalized performance plans. Data driven training programs and rehabilitation protocols.	No		Not stated	Kinduct	Yes
Mobile application	Metrifit	Descriptive analytics and intelligent feedback for altering coaches and athletes and behavioral changes. Body and mind module (mood state, sleep quality and duration, energy levels, health, muscle readiness, nutrition, stress). Session RPE module Injury tracker. Analytics/reports. Daily Traffic Light report for coaches on their athletes. Team training load report. Acute:Chronic workload ratio.	No		Not stated	Metrifit	Yes
Mobile application	Athlete Monitoring	Mobile application Recovery, risk and readiness monitoring (soreness, stress, health, sleep). Pre-training wellness questionnaire (sleep quality, stress, fatigue, heart rate variability). Record, import, store and track data. Customizable questionnaires. Injury tracking and health management (Scat3 concussion assessment; mental health survey; eating disorder screening; depression screening; alcohol use; sleep apnoea). Data import from wearable devices (e.g., GPS and HR). Alerts and dashboards are updated in real-time using individual planned and reported data. Evidence-based algorithms are used to detect issues.	No		Not stated	Fitstats Technologies Inc	Yes
Mobile application	SportsMed Elite	Offers predictive insights into sports, wellness and performance data. Psychological data; fatigue, motivation, stress. Physical data; muscle tightness and soreness. Nutrition; appetite, nutrition quality. Technology; phone use before bed. Readiness; general soreness, illness, recovery, productivity. Sleep; quality, quantity. Capacity for recording data on anthropometry, performance tests, injury, and rehabilitation.	No		Not stated	SMG Technologies	Yes
Mobile application	SMARTABASE	Records data on injury and rehabilitation, training loads, performance metrics for predictive purposes and talent identification. Records data on muscle soreness, stress/pressure, sleep, types, and amount of physical activity. Direct connections with 3rd party products.	No		Not stated	Fusion Sport	Yes

Among these devices listed in Table 2, the I Measure U device is lightweight, compact and offers the greatest versatility. Other devices and garments provide information about muscle activation and basic training metrics (e.g., steps, speed, distance, cadence, strokes, repetitions etc). The mPower is a pod placed on the skin that measures EMG. It provides a simple, wireless alternative to more complex EMG equipment. Likewise, the Athos garments contain EMG sensors, but the garments have not been properly validated. It is debatable whether the Sensoria and Dynafeed garments offer any more benefits than other devices. The Mettis Trainer insoles (and Arion insoles in development) could provide some useful feedback on running biomechanics in the field. None of these devices have been independently tested to determine their validity or reliability. Until such validity and reliability data become available, these devices should (arguably) be used in combination with more detailed motion-capture video analysis.

Various mobile applications have been developed for recording and analyzing training loads and injury records (Table 2). These applications include a wide range of metrics that incorporate aspects of both physical and psychological load. The Metrifit application provides users with links to related unpublished research on evaluating training stress. Many of the applications record and analyze similar metrics, so it is difficult to differentiate between them. The choice of one particular application will most likely be dictated by individual preferences. With such a variety of metrics—which are generally recorded indirectly—it is difficult to perform rigorous validation studies on these products. Another limitation of some of these applications is the large amount of data they record and how to make sense of all the data.

## Technologies for monitoring heart rate, heart rate variability, and breathing patterns

Various devices and mobile applications have been developed for monitoring physiological stress and workloads during exercise (Table 3). The devices offer some potential advantages and functionality over traditional heart rate monitors to assess demands on the autonomic nervous system and the cardiovascular system during and after exercise. They can therefore be used by athletes, soldiers and workers involved in physically demanding jobs (e.g., firefighters) to monitor physical strain while they exercise/work, and to assess when they have recovered sufficiently.

**Table 3:** Devices and garments for monitoring cardiorespiratory functions.

**Product category**	**Product name**	**Technical characteristics**	**Validated**	**Reliability testing**	**Calibration required**	**Development company/organization**	**Commercially available**
Smart watch	HELO	Monitors blood pressure, heart rate, ECG, blood temperature and O_2_ saturation, sleep cycle, breathing rate, calories, mood, and physical activity levels. Germanium, Hematite and Himalayan Salt plates to improve blood circulation, eliminate toxins, and purify cells.	No	No	Not stated	HELO	Yes
Smart watch	E4 Wristband	Contains a photoplethysmography sensor that records blood pulse volume (from which heart rate and heart rate variability can be derived) a 3-axis accelerometer for recording activity an electrodermal sensor to measure activity of the sympathetic nervous system (to derive features related to stress, engagement, and excitement) an infrared thermophile to record skin temperature. Connected to a mobile application and data stored in a cloud.	No	No	Uncertain	Empatica Inc	Yes
Smart watch	Reign Active Recovery Band	Records type and amount of activity, calories burned, heart rate variability (through two metal sensors). Calculates a “Go-Zone” based on heart rate variability to determine personal fatigue and recovery. Training recommendations based on heart rate variability. Records habitual sleep patterns (through an accelerometer) to determine personal “Ideal Sleep” hours; makes recommendations for sleep Connects to mobile application.	No	No	Uncertain	Jaybird	Yes
Smart watch	Amiigo	Monitors heart rate, heart rate variability, blood pressure variations, pulse volume variations, respiratory rate, skin temperature, arterial blood O_2_ saturation, sleep time/quality, restful sleep, calories burned. Connected to mobile application.	No	No	Not stated	Amiigo	Yes
Smart watch	Mio SLICE™	Monitors physical activity levels and heart rate. Calculates Personal Activity Intelligence (PAI) score to match physical activity and heart rate to health assessment.	No	No	Not stated	Mio™	Yes
Strap/band/patch	Lief	Patch that monitors heart rate and breathing rate. Provides haptic signals to the user following extended periods of stress. Associated mobile application records various emotions to create a mood rating and provides cognitive behavioral therapy for emotional regulation.	No	No	No	Lief Therapeutics	Not at present
Strap/band/patch	Zephyr™	Sensor connected to a strap around the chest or imbedded within a singlet. Measures physiological data including heart rate, breathing rate, heart rate variability, estimated body temperature, calories burned, blood pressure, arterial blood O_2_ saturation.	No	No	Uncertain	Medtronic	Yes
Strap/band/patch	Biostrap	Wristband that captures high-fidelity raw photoplethysmography waveforms to evaluate heart health. Connected to mobile application.	No	No	No	Biostrap USA, LLC	Yes
Wearable device	CorSense HRV monitor	Portable device placed on the finger and connected to a mobile application to measure heart rate variability, provide a readiness score, guide to stress and recovery.	No	No	Uncertain	CorSense	Not at present
Non-wearable device	MyCalmBeat	Near infrared pulse meter to assess personal best breathing rate when calm and train breathing at that rate. Consciously monitoring and adjusting breathing rate improves heart rate variability, leading to greater resilience, better pain management, improved sense of wellbeing, enhanced ability to focus and think clearly. Connected to mobile application.	No	No	Self-calibration	MyBrainSolution	
Garment	Hexoskin	Singlet garment containing an ECG sensor, a breathing sensor and an accelerometer; measures: heart rate, heart rate variability, breathing rate, tidal volume, minute ventilation, steps, cadence, estimated calories burned. Connected to mobile application.	Yes	Yes	Not stated	Carre Technologies Inc (Hexoskin) ©	Yes
Mobile application and non-wearable device	OmegaWave	Evaluates heart rate variability, neuromuscular, sensorimotor, and physical work capacity. Data derived to determine Windows of Trainability™ for “readiness” of central nervous, cardiac, energy supply, gas exchange/pulmonary and hormonal systems and detoxification. Sensors placed on the body to record ECG and DC potential. Team and individual athletes analysis packages.	No	No	Uncertain	OmegaWave	Yes

Among the devices listed in Table 3, the OmegaWave offers the advantages that it directly records objective physiological data such as the electrocardiogram (ECG) as a measure of cardiac stress and direct current (DC) potential as a measure of the activity of functional systems in the central nervous system. However, one limitation of the OmegaWave is that some of the data it provides (e.g., energy supply, hormonal function, and detoxification) are not measured directly. Accordingly, the validity and meaningfulness of such data is uncertain.

The Zephyr sensor, E4 wristband and Reign Active Recovery Band offer a range of physiological and biomechanical data, but these devices have not been validated independently. The E4 wristband is also very expensive for what it offers. The Mio SLICE™ wristband integrates heart rate and physical activity data with an algorithm to calculate the user's Personal Activity Intelligence score. Over time, the user can employ this score to evaluate their long-term health status. Although this device itself has not been validated, the Personal Activity Intelligence algorithm has been tested in a clinical study (Nes et al., 2017). The results of this study demonstrated that individuals with a Personal Activity Intelligence score ≥100 had a 17–23% lower risk of death from cardiovascular diseases.

The HELO smart watch measures heart rate, blood pressure, and breathing rate. It also claims to have some more dubious health benefits, none of which are supported by published or peer-reviewed clinical studies. One benefit of the HELO smart watch is that it can be programmed to deliver an emergency message to others if the user is ill or injured.

The Biostrap smart watch measures heart rate. Although it has not obviously been validated, the company provides a link to research opportunities using their products, which suggests confidence in their products and a willingness to engage in research. The Lief patch measures stress levels through heart rate variability (HRV) and breathing rate, and provides haptic feedback to the user in the form of vibrations to adjust their emotional state. The option of real-time feedback without connection to other technology may provide some advantages. If worn continuously, it is uncertain if or how this device (and others) distinguishes between changes in breathing rate and HRV associated with “resting” stress, as opposed to exercise stress (Dupré et al., 2018). But it is probably safe to assume that users will be aware of what they are doing (i.e., resting or exercising) during monitoring periods. Other non-wearable equipment is available for monitoring biosignals relating to autonomic function and breathing patterns. MyCalmBeat is a pulse meter that attaches to a finger to assess and train breathing rate, with the goal of improving emotional control. The CorSense HRV device will be available in the future, and will be tailored for athletes by providing a guide to training readiness and fatigue through measurements of HRV. It is unclear how data from these devices compare with applications such as OmegaWave, which measures ECG directly vs. by photoplethysmography.

A range of garments with integrated biosensor technology have been developed. The Hexoskin garment measures cardiorespiratory function and physical activity levels. It has been independently validated (Villar et al., 2015). The device demonstrates very high agreement with heart rate measured by ECG (intraclass correlation coefficient >0.95; coefficient of variation <0.8%), very high agreement with respiration rate measured by turbine respirometer (intraclass correlation coefficient >0.95; coefficient of variation <1.4%), and moderate to very high agreement with hip motion intensity measured using a separate accelerometer placed on the hip (intraclass correlation coefficient 0.80 to 0.96; coefficient of variation <6.4%). This device therefore offers value for money. Other garments including Athos and DynaFeed appear to perform similar functions and are integrated with smart textiles, but have not been validated.

## Technologies for monitoring and promoting better sleep

Many devices have been designed to monitor and/or promote sleep (Table 4). Baron et al. (2017) have previously published an excellent review on these devices. Sleep technologies offer benefits for anyone suffering sleep problems arising from chronic disease (e.g., sleep apnea), anxiety, depression, medication, travel/work schedules, and environmental factors (e.g., noise, light, ambient temperature). The gold standard for sleep measurement is polysomnography. However, polysomnography typically requires expensive equipment and technical expertise to set up, and is therefore not appropriate for regular use in a home environment.

**Table 4:** Wearable devices and equipment for monitoring and promoting better sleep.

**Product category**	**Product name**	**Technical characteristics**	**Validated**	**Reliability testing**	**Calibration required**	**Development company/organization**	**Commercially available**
Wearable device	UP™	Wristband connected to a mobile application. Activity tracker to measure light, deep and rapid eye movement sleep. Measures heart rate.	Yes	No	No	Jawbone	Yes
Wearable device	FitBit Flex™	Wristband connected to a mobile application. Activity tracker to total sleep time, time in bed.	No	Yes	No	FitBit	Yes
Wearable device	FitBit Charge2™	Wristband connected to a mobile application. Activity tracker to total sleep time, time in bed.	Yes	No	No	FitBit	Yes
Wearable device	OURA	Ring with 3D accelerometer and gyroscope to measure light, deep, and rapid eye movement sleep. Measures heart rate.	Yes	No	No	OURA	No
Wearable device	Dreem	Headband that transmits sound simulations through bone conduction technology that synchronize with sleep. Miniaturized EEG sensors provide feedback on sleep through mobile application.	No	No	No	Rythm	Yes
Wearable device	Plex® Sleep Scanner	Chest strap that measures breathing patterns, pulse and oxygen levels during sleep. Connects to mobile application.	No	No	Not stated	Somnology	No
Wearable device	Sleep Profiler PSG2	EEG sleep monitor. Three channels of frontal EEG. Pulse rate and optional ECG. Monitors head movement and position. Provides data on total time and percentage sleep, rapid eye movement and slow wave sleep, sleep efficiency and average number of cortical, sympathetic and behavioral arousals. Recording device connects to computer to download data.	No	No	No	Advanced Brain Monitoring	Yes
Wearable device	Zmachine®	Three skin sensors placed behind each ear and the back of the neck are connected to a device for recording EEG. Records periods of light sleep, deep sleep, rapid eye movement, arousals, sleep period time, total sleep time, sleep efficiency, latency to sleep persistency, wake after sleep onset and time spent out of bed. Recording device connects to computer to download data. Two models (Insight and Synergy) available.	No	No	No	General Sleep Corporation	Yes
Wearable device	Somté PSG	Headband device with 6-channel EEG for polysomnography (PSG) assessment. Enable to simultaneously record oculomotor activity and ECG. Bluetooth wireless connection to computer software for sleep staging and events.	No	No	No	Compumedics®	Yes
Wearable device	Sleep Shepherd	Fabric headband that monitors EEG signals and sends audio sounds to reduce brain activity to a level conducive to sleep. Mobile application tracks sleep and provides alarm to lift brain out of sleep before the user wakes up.	No	No	No	Sleep Shepherd LLC	Yes
Wearable device	Re-Timer	Eyewear that projects green-blue light. Designed to be worn for 30 min in morning or afternoon. Used to re-train timing of sleep onset. Online calculator available for sleep schedules and adjustment to jet lag.	No	No	No	Re-Time Pty Ltd	Yes
Wearable device	AYO	Eyewear containing sensors to detect ambient light and projects blue light. Connected to mobile application to deliver blue light at the best time of day or night according to personal preferences and lifestyle (e.g., known periods of sleepiness or low energy); programmable to match different time zones.	No	No	No	Novology	Yes
Wearable device	illumy Sleep Smart Mask	Mask that uses gently dimming red light to promote sleep and gently brightening blue light to wake up. Sleep and wake times programmed into mobile application and synched to mask.	No	No	No	Headwaters Inc	Yes
Wearable device	HUSH	Wireless ear plugs connected to a mobile application that plays soothing music to encourage sleep or wakefulness.	No	No	No	Hush technology Inc	Not at present
Wearable device	Kokoon	Headphones that mold to the shape of the user's head. Detects EEG signals and movement to find the lightest point of the user's natural sleep cycle during which to wake up. Active noise cancellation and white noise.	No	No	Not stated	Kokoon	Not at present
Non-wearable device	Dreampad	Specialized pillow connected to a mobile application with programmable songs designed to encourage relaxation and sleep. Music is relayed through the pillow.	No	No	No	Dreampad	Yes
Non-wearable device	NightWave Sleep Assistant	Device that projects a soft blue light. Slow steady breathing coupled with blue light is intended to promote onset of sleep.	No	No	no	NightWave®	Yes
Non-wearable device	Withings Aura and REM Sleep Tracker	Light-emitting diodes that project light of different colors to promote sleep or wakefulness. Programmable music to accompany time of waking. Sensors to detect ambient temperature, light intensity, and sound. Optional sleep sensor placed under mattress to monitor sleep duration, sleep cycle (light, deep, rapid eye movement), time awake; wakes you up at best time of the sleep cycle.	No	No	No	Nokia	Yes
Non-wearable device	Circadia sleep tracker	Contactless sensor that attaches to bedroom wall. Wireless measurement of heart rate, breathing, and body movement while sleeping. Integrated environmental sensors detect ambient temperature, humidity, light, and sound. Sleeping patterns calculate a model of the body's internal clock, signaling when your body will be at peak alertness, when you'll start feeling tired and when your body is ready to sleep. Model also predicts how much your internal clock is out of sync, and the impact on your alertness and sleep quality later in the day.	No	No	Not stated	Circadia	No
Non-wearable device	Beddit3 Sleep Tracker	Device with pressure, capacitive touch, humidity, and temperature sensors; placed under the mattress. Connected to mobile application records sleep time, sleep efficiency, time to fall asleep, restless sleep, sleep cycles, light/deep sleep, bedtime, wake-up time, away from bed, awake in bed, sleep score, heart rate and breathing cycles. No wearable devices required.	No	No	No	Beddit	Yes
Non-wearable device	ResMed+	“Non-contact” device for monitoring sleep, ambient temperature, light, and noise. Projects soothing sounds to promote sleep onset. Connected to mobile application that provides data on different sleep cycles, sleep patterns and a smart alarm to wake the user during light sleep. Measures breathing and movement patterns.	No	No	No	ResMed	Yes

The Advanced Brain Monitoring Sleep Profiler and Zmachine Synergy have been approved by the US Food and Drug Administration. Both devices monitor various clinical metrics related to sleep architecture, but both are also quite expensive for consumers to purchase. The disposable sensor pads required to measure encephalogram (EEG) signals add an extra ongoing cost. The Somté PSG device offers the advantage of Bluetooth wireless technology for recording EEG during sleep, without the need for cables.

A large number of wearable devices are available that measure various aspects of sleep. Several of these devices have been validated against gold-standard polysomnography. The UP™ and Fitbit Flex™ devices are wristbands connected to a mobile application. One study reported that compared with polysomnography, the UP device has high sensitivity for detecting sleep (0.97), and low specificity for detecting wake (0.37), whereas it overestimates total sleep time (26.6 ± 35.3 min) and sleep onset latency (5.2 ± 9.6 min), and underestimates wake after sleep onset (31.2 ± 32.3 min) (de Zambotti et al., 2015). Another study reported that measurements obtained using the UP device correlated with total sleep time (*r* = 0.63) and time in bed (*r* = 0.79), but did not correlate with measurements of deep sleep, light sleep or sleep efficiency (Gruwez et al., 2017). Several studies have reported similar findings for the Fitbit Flex™ device (Montgomery-Downs et al., 2012; Mantua et al., 2016; Kang et al., 2017). In a validation study of the OURA ring, it was shown to record similar total sleep time, sleep latency onset and wake after sleep onset, and had high sensitivity for detecting sleep (0.96). However, it had lower sensitivity for detecting light sleep (0.65), deep sleep (0.51) and rapid eye movement sleep (0.61), and relatively poor specificity for detecting wake (0.48). It also underestimated deep sleep by about 20 min, and overestimated the rapid eye movement sleep stage of sleep by about 17 min (de Zambotti et al., 2017b). Similar results were recently reported for the Fitbit Charge2™ device (de Zambotti et al., 2017a). These devices therefore offer benefits for monitoring some aspects of sleep, but they also have some technical deficiencies.

Various other devices are available that play soft music or emit light of certain colors to promote sleep or wakefulness. Some similar devices are currently in commercial development. Although devices such as the Withings Aura and REM Sleep Tracker, Re-Timer and AYO have not been independently validated, other scientific research supports the benefits of applying blue light to improve sleep quality (Viola et al., 2008; Gabel et al., 2013; Geerdink et al., 2016). The NightWave Sleep Assistant is appealing based on its relatively low price, whereas the Withings Aura and REM Sleep Tracker records sleep patterns. The Re-Timer device is useful based on its portability.

Some devices also monitor temperature, noise and light in the ambient environment to identify potential impediments to restful sleep. The Beddit3 Sleep Tracker does not require the user to wear any equipment. The ResMed S+ and Circadia devices are entirely non-contact, but it is unclear how they measure sleep and breathing patterns remotely.

## Technologies for monitoring psychological stress and evaluating cognitive function

The nexus between physiological and psychological stress is attracting more and more interest. Biofeedback on emotional state can assist in modifying personal appraisal of situations, understanding motivation to perform, and informing emotional development. This technology has application for monitoring the health of people who work under mentally stressful situations such as military combat, medical doctors, emergency service personnel (e.g., police, paramedics, fire fighters) and traffic controllers. Considering the strong connection between physiology and psychology in the context of competitive sport, this technology may also provide new explanations for athletic “underperformance” (Dupré et al., 2018).

Technology such as the SYNC application designed by Sensum measures emotions by combining biometric data from third-party smartwatches/wristbands, medical devices for measuring skin conductance and HR and other equipment (e.g., cameras, microphones) (Dupré et al., 2018). The Spire device is a clip that attaches to clothing to measure breathing rate and provide feedback on emotional state through a mobile application. Although this device has not been formally validated in the scientific literature, it was developed through an extended period of university research. The Feel wristband monitors emotion and provides real-time coaching about emotional control.

In addition to the mobile applications and devices that record and evaluate psychological stress, various applications and devices have also been developed to measure EEG activity and cognitive function (Table 5). Much of this technology has been extensively engineered, making it highly functional. Although the technology has not been validated against gold standards, there is support from the broader scientific literature for the benefits of biofeedback technology for reducing stress and anxiety (Brandmeyer and Delorme, 2013). The Muse™ device produced by InterAxon is an independent EEG-biofeedback device itself, but it has also been coupled with other biofeedback devices and mobile applications (e.g., Lowdown Focus, Opti Brain™). The integration of these technologies highlights the central value of measuring EEG and the versatility of the Muse™ device. The NeuroTracker application is based around the concept of multiple object tracking, which was established 30 years ago as a research tool (Pylyshyn and Storm, 1988). NeuroTracker has since been developed as a training tool to improve cognitive functions including attention, working memory, and visual processing speed (Parsons et al., 2016). This technology has potential application for testing and training cognitive function in athletes (Martin et al., 2017) and individuals with concussion (Corbin-Berrigan et al., 2018), and improving biological perception of motion in the elderly (Legault and Faubert, 2012). The NeuroTracker application has not been validated.

**Table 5:** Wearable devices and mobile applications for monitoring psychological stress, brain activity, and cognitive function.

**Product category**	**Product name**	**Technical characteristics**	**Validated**	**Reliability testing**	**Calibration required**	**Development company/ organization**	**Commercially available**
Mobile application	Opti Brain™	Coupled to Muse™ headband for tracking brain activity while performing different tasks. Maps and displays patterns of activity in four areas of the brain. Also offers advanced option to analyse EEG maps.	No	No	Self-calibration	Opti Brain	Yes
Mobile application	T2 Mood Tracker	Monitors and tracks emotional health. Records a range of emotions for anxiety, depression, head injury, stress, posttraumatic stress, and general well-being. Tracks progress in customizable areas and displays results in an easy-understand graph.	No	No	Not stated	National Center for Telehealth and Technology	Yes
Task-based mobile application	King-Devick Test	Test cognitive function and eye movement under healthy conditions and following a concussion.	Yes	Yes	Baseline screening recommended		Yes
Task-based mobile application	HitCheck	Tests short term memory, balance, coordination, visual memory, impulse control, long term memory, reaction time, problem solving and color recognition,	No	No	Not stated	HitCheck	Yes
Task-based mobile application	BrainCheck Sport™	Tests attention, memory, response time and visual processing.	No	No	Not stated	BrainCheck	Yes
Task-based mobile application	BrainFx	Assessment of mild brain disorders. Measures up to 30 cognitive function skills, including mood, social, behavioral, fine motor and balance effects. Two platforms available to provide different levels of assessment. Requires training as an assessor.	No	No	No	BrainFx	Yes
Task-based mobile application	Sway	Tests balance and reaction time, and tracking symptom severity (e.g., headache, neck pain, nausea, vomiting, dizziness, blurred vision, sensitivity to light).	Yes	No	Not stated	Sway Medical LLC	Yes
Task-based computer software/mobile application	NeuroTracker	Computer software that uses 3D multiple object tracking at increasing difficulties to develop high-level brain functions. Includes a series of mini-tests that involves remembering key targets, tracking them among moving distractors and then identifying them. Intended to improve attention and executive function, increase brainwave and processing speed, inhibition and response control, increase biological motion perception, filter out distractions, make more tactical and accurate decisions and improve anticipation and response times.	No	No	Uncertain	CogniSens Inc	Yes
Task-based computer software	HeadSmart™	Computer programme to assess simple reaction time, learning and memory skills, attention, and concentration, problem solving.	No	No	Baseline screening recommended	HeadSmart™ Sport Concussion Programme	Yes
Strap/band/patch	Feel	Wristband connected to a mobile application that monitors emotions and offers training for emotional regulation.	No	No	No	Sentio Solutions Inc	Not at present
Strap/band/patch	CSx	A microsensor that detects linear and rotational acceleration forces exerted on the head during collisions. Connected to a mobile application.	No	No	No	CSx	Uncertain
Strap/band/patch	Triax™	A triaxial microsensor that detects acceleration forces exerted on the head. Worn attached to a headband or a skullcap. Connected to a mobile application.	No	No	No	Triax Technologies Inc	Yes
Strap/band/patch	X-Patch Pro	A sensor that attaches behind the ear, records impacts forces and sends data to a mobile application.	No	No	No	X2 Biosystems Inc	Yes
Wearable device	Prevent™ mouthguard	Custom fabricated or individually molded mouthguards with sensors to monitor impact forces with 6 degrees of freedom. Patented algorithm calculates center of gravity of the head and measures the force, location and direction of each head impact. Compares each impact to a pre-set Max G head impact threshold. Data uploaded via the cloud to a mobile application for monitoring by medical staff.	No	No	Not stated	Prevent™ Concussion Intelligence	Yes
Wearable device	Spire	Clip that attaches to clothing to monitor breathing rate, provides feedback on emotional state and recommendations for controlling stress.	No	No	No	Spire	Yes
Wearable device	Muse™	Headband that measures EEG. Biofeedback provided to control breathing pattern to reduce brain activity and stimulate relaxation. Reduces stress and anxiety and improves resilience. Personal data record and training goals.	No	No	Self-calibration	InterAxon	Yes
Wearable device	Lowdown Focus	Sports/fashion eyewear with EEG sensors embedded in the ear bridges. Coupled with a variation of the Muse™ mobile application. Provides real-time feedback on brain activity and cognitive training activities to improve focus, decision-making, relaxation, attention and emotional control.	No	No	Self-calibration	Smith	Yes
Wearable device	PortaLite	Small, lightweight, flexible portable single-channel oxygenation monitoring device. Uses near infrared spectroscopy to measure oxy-, deoxy- and total hemoglobin concentrations at capillary level. Capable of measuring tissue saturation index. Used for monitoring task specific cerebral oxygenation levels particularly during real world activities. Bluetooth (150 m) or on board data collection.	Independent published research	No	Yes	Artinis Medical Systems	Yes
Wearable device	OctaMon	Lightweight, flexible portable 8-channel oxygenation monitoring device. Uses near infrared spectroscopy to measure oxy-, deoxy- and total hemoglobin concentrations at capillary level. Used for monitoring task specific cerebral oxygenation levels. Bluetooth (100 m) data collection; real-time data analysis.	Independent published research	No	Yes	Artinis Medical Systems	Yes
Wearable device	Brite23	Portable 23 channel, lightweight (<300 g) fNIRS device. Uses near infrared spectroscopy to measure oxy-, deoxy- and total hemoglobin concentrations at capillary level. Used for monitoring cerebral oxygenation levels during real-world activities including everyday physical activities and sports exercises. Bluetooth (30 m) data collection; real-time data analysis. Fully synchronized with other physiological and behavioral measurements; integration with multiple NIRS devices within a single data stream. Offline measurement data storage for over 200 h of recording.	Independent published research.	Yes	Yes	Artinis Medical Systems	Yes
Wearable device	NIRSPORT	Portable 8-channel near infrared spectroscopy for recording cerebral oxy- and deoxy- hemoglobin concentrations. Wireless real-time data streaming. Enabled to couple with other measurements of EEG, EMG, functional magnetic resonance imaging, eye-tracking.	Independent published research	No	Yes	NIRx	Yes
Wearable device	Mobita	Portable wireless 32 channel physiological signal amplifier for EEG, EMG and other physiological or biomechanical data. Built-in 3D accelerometer. True active shielding for all channels. Battery operated with multiple channel configurations. Real time wireless (10 m) or flash disk recording (16 GB). 2 kHz sampling per channel and 24 bit data resolution.	Independent published research	No	Yes	TMSi	Yes
Wearable device	g.Nautilus	Portable wireless 32 channel EEG. Active electrodes. 3D accelerometer. 24 bit data resolution, 500 Hz sampling rate. Real time wireless (10 m).	Independent published research	No	Yes	G.Tec Medical Engineering	Yes
Wearable device	Starstim fNIRS	Headset that combines sensors for measuring EEG with near infrared sensors for local blood blow (hemodynamics). Is also capable of applying transcranial direct stimulation. Application for understanding cognitive function.	Independent published research		Yes	Artinis Medical Systems	Yes
Wearable device	B2v2	Headband containing sensors that read EEG signals that are then converted to audio sounds in headphones. By listening to the sounds, the brain recognizes imbalances and recalibrates itself to create more balanced brainwaves. Intended to improve stress management, memory, self-awareness, mental flexibility, and quality of sleep.	No	No	Not stated	Brain State Technologies	Yes
	EyeSinc®	Device for measuring oculomotor activity following a concussion.	No	No	Not stated	SyncThink	No

In the fields of human factors and ergonomics, there is increasing interest in methods to assess cognitive load. Understanding cognitive load has important implications for concentration, attention, task performance, and safety (Mandrick et al., 2016). The temporal association between neuronal activity and regional cerebral blood flow (so-called “neurovascular coupling”) is recognized as fundamental to evaluating cognitive load. This assessment is possible by combining ambulatory functional neuroimaging techniques such as EEG and functional near infrared spectroscopy (fNIRS) (Mandrick et al., 2016). Research exists on cognitive load while walking in healthy young and older adults (Mirelman et al., 2014; Beurskens et al., 2016; Fraser et al., 2016), but there does not appear to be any research to date evaluating cognitive load in athletes. A number of portable devices listed in Table 5 measure fNIRS, and some also measure EEG and EMG. These integrated platforms for measuring/assessing multiple physiological systems present significant value for various applications. These devices all measure physiological signals directly from the brain and other parts of the body. Research using these devices has demonstrated agreement between measurements obtained from fNIRS vs. the gold standard of functional magnetic resonance imaging (Mehagnoul-Schipper et al., 2002; Huppert et al., 2006; Sato et al., 2013; Moriguchi et al., 2017). These devices require some expertise and specialist training.

Concussion is a common occurrence in sport, combat situations, the workplace, and in vehicular accidents. There is an ever-growing need for simple, valid, reliable, and objective methods to evaluate the severity of concussion, and to monitor recovery. A number of mobile applications and wearable devices have been designed to meet this need. These devices are of potential value for team doctors, physical trainers, individual athletes, and parents of junior athletes.

The King-Devick Test® is a mobile application based on monitoring oculomotor activity, contrast sensitivity, and eye movement to assess concussion. It has been tested extensively in various clinical settings, and proven to be easy to use, reliable, valid, sensitive, and accurate (Galetta et al., 2011; King et al., 2015; Seidman et al., 2015; Walsh et al., 2016). Galetta et al. (2011) examined the value of the King-Devick Test® for assessing concussion in boxers. They discovered that worsening scores for the King-Devick Test® were restricted to boxers with head trauma. These scores also correlated (ρ = 90; *p* = 0.0001) with scores from the Military Acute Concussion Evaluation, and showed high test–retest reliability (intraclass correlation coefficient 0.97 [95% confidence interval 0.90–1.0]). Other studies have reported a very similar level of reliability (King et al., 2015). Performance in the King-Devick Test® is significantly impaired in American football players (Seidman et al., 2015), rugby league players (King et al., 2015), and combat soldiers (Walsh et al., 2016) experiencing concussion. Because the King-Devick Test® is simple to use, it does not require any medical training, and is therefore suitable for use in the field by anyone.

The EyeSync® device employs a simple test that records eye movement during a 15-s circular visual stimulus, and provides data on prediction variability within 60 s. It is not yet commercially available, and has therefore not been validated. The BrainCheck Sport™ mobile application employs the Flanker and Stroop Interference test to assess reaction time, the Digit Symbol Substitution test to evaluate general cognitive performance, the Trail Making test to measure visual attention and task switching, and the Coordination test. It has not been independently validated, but is quick and uses an array of common cognitive assessment tools.

The Sway mobile application tests balance and reaction. Its balance measurements have been validated in small scale studies (Patterson et al., 2014a,b). Performance in the Sway test was inversely correlated (*r* = −0.77; *p* < 0.01) with performance in the Balance Error Scoring System test (Patterson et al., 2014a) and positively correlated (*r* = 0.63; *p* < 0.01) with performance in the Biodex Balance System SD (Patterson et al., 2014b). Further testing is needed to confirm these results. One limitation of this test is the risk of bias that may occur if individuals intentionally underperform during baseline testing to create lower scores than they may attain following a concussion (so as to avoid time out of competition after concussion).

Various microsensors have been developed for measuring impact forces associated with concussion (Table 5). Some of these microsensors attach to the skin, whereas others are built into helmets, headwear or mouth guards. The X-Patch Pro device is a device that attaches behind the ear. Although it has not been scientifically validated against any gold standard, it has been used in published concussion research projects (Swartz et al., 2015; Reynolds et al., 2016), which supports its sensitivity for assessing head impact forces. The Prevent™ mouth guard is a new device for measuring the impact of head collisions. Its benefits include objective and quantitative data on the external force applied to the head. Many of the sensors vary in accuracy, and only record linear and rotational acceleration. Whereas, many sports involve constantly changing of direction, planes of movement will provide the most accurate data. A study by Siegmund et al. (2016) reported that the Head Impact Telemetry System (HITS) sensors detected 861 out of the 896 impacts (96.1%). If a sensor is detecting better than 95%, it has good reliability. However, helmetless sports have fewer options for such accuracy and actionable data.

## Considerations and recommendations

In a brief, yet thought-provoking commentary on mobile applications and wearable devices for monitoring sleep, Van den Bulck makes some salient observations and remarks that are applicable to all forms of consumer health technologies (Van den Bulck, 2015). Most of these technologies are not labeled as medical devices, yet they do convey explicit or implicit value statements about our standard of health. There is a need to determine if and how using technology influences peoples' knowledge and attitude about their own health. The ever-expanding public interest in health technologies raises several ethical issues (Van den Bulck, 2015). First, self-diagnosis based on self-gathered data could be inconsistent with clinical diagnoses provided by medical professionals. Second, although self-monitoring may reveal undiagnosed health problems, such monitoring on a large population level is likely to result in many false positives. Last, the use of technologies may create an unhealthy (or even harmful) obsession with personal health for individuals or their family members who use such technologies (Van den Bulck, 2015). Increasing public awareness of the limitations of technology and advocating health technologies that are both specific and sensitive to certain aspects of health may alleviate these issues to some extent, but not entirely.

**Figure 2:**
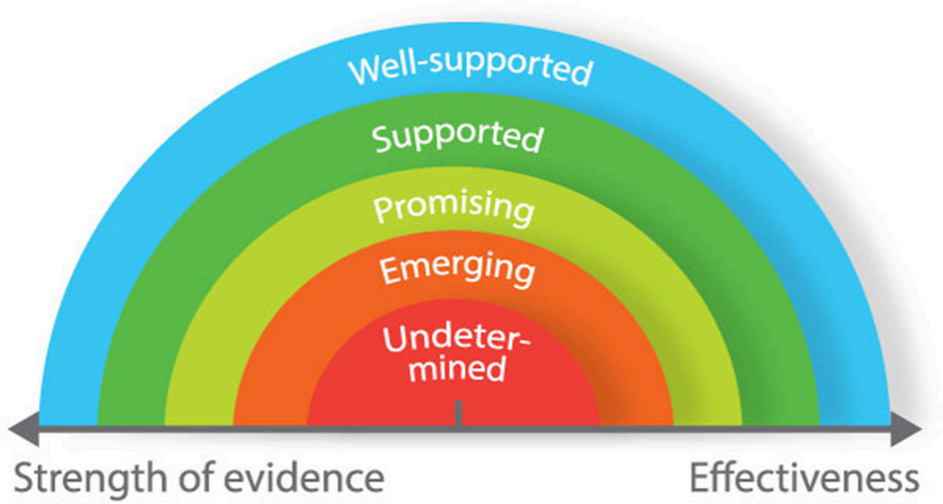
Matrix to guide decision-making process for evaluating and selecting new technologies.

For consumers who want to evaluate technologies for health and performance, we propose a matrix based around two dimensions: strength of evidence (weak to strong) and effectiveness (low to high) (Figure 2). This matrix is based on a continuum that was developed for use in a different context (Puddy and Wilkins, 2011), but is nonetheless appropriate for evaluating technology. When assessing the strength of evidence for any given technology, consumers should consider the following questions: (i) how rigorously has the device/technology been evaluated? (ii) how strong is the evidence in determining that the device/technology is producing the desired outcomes? (iii) how much evidence exists to determine that something other than this device/technology is responsible for producing the desired outcomes? When evaluating the effectiveness of technologies, consumers should consider whether the device/technology produces desirable or non-desirable outcomes. Applying the matrix in Figure 2, undetermined technologies would include those that have not been developed according to any real-world need and display no proven effect. Conversely, well-supported technologies would include those that have been used in applied studies in different settings, and proven to be effective.

**Figure 3:**
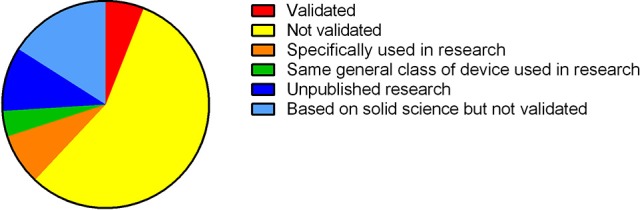
Classification of technologies based on whether they have been validated and/or used in research.

Most of the health and performance technologies that we have reviewed have been developed based on real-world needs, yet only a small proportion has been proven effective through rigorous, independent validation (Figure 3). Many of these technologies described in this review should therefore be classified “emerging” or “promising.” Independent scientific validation provides the strongest level of support for technology. However, it is not always possible to attain higher standards of validation. For example, cognitive function is underpinned by many different neurological processes. Accordingly, it is difficult to select a single neurological measurement to compare against. Some technologies included in this review have not been independently validated *per se*; but through regular use in academic research, it has become accepted that they provide reliable and specific data on measurement items of interest. Even without formal independent validation, it is unlikely (in most instances at least) that researchers would continue using such technologies if they did not offer reliable and specific data. In the absence of independent validation, we therefore propose that technologies that have not been validated against a gold standard (but are regularly used in research) should be considered as “well-supported.” Other technical factors for users to consider include whether the devices require calibration or specialist training to set up and interpret data, the portability and physical range for signal transmission/recording, Bluetooth/ANT+ and real-time data transfer capabilities, and on-board or cloud data storage capacity and security.

From a research perspective, consumer health technologies can be categorized into those that have been used in validation studies, observational studies, screening of health disorders, and intervention studies (Baron et al., 2017). For effective screening of health disorders and to detect genuine changes in health outcomes after lifestyle interventions, it is critical that consumer health technologies provide valid, accurate and reliable data (Van den Bulck, 2015). Another key issue for research into consumer health technologies is the specificity of study populations with respect to the intended use of the technologies. If technologies have been designed to monitor particular health conditions (e.g., insomnia), then it is important for studies to include individuals from the target population (as well as healthy individuals for comparison). Scientific validation may be more achievable in healthy populations compared with populations who have certain health conditions (Baron et al., 2017). There is some potential value for commercial technology companies to create registries of people who use their devices. This approach would assist in collecting large amounts of data, which would in turn provide companies with helpful information about the frequency and setting (e.g., home vs. clinic) of device use, the typical demographics of regular users, and possible feedback from users about devices. Currently, very few companies have established such registries, and they are not consistently publishing data in scientific journals. Proprietary algorithms used for data processing, the lack of access to data by independent scientists, and non-random assignment of device use are also factors that are restricting open engagement between the technology industry and the public at the present time (Baron et al., 2017).

It would seem advisable for companies producing health and performance technologies to consult with consumers to identify real-world needs and to invest in research to prove the effectiveness of their products. However, this seems to be relatively rare. Budget constraints may prevent some companies from engaging in research. Alternatively, some companies may not want to have their products tested independently out of a desire to avoid public scrutiny about their validity. In the absence of rigorous testing, before purchasing health and performance technologies, consumers should therefore carefully consider whether such technologies are likely to be genuinely useful and effective.

## Author contributions

JP and JS conceived the concept for this review. JP, GK, and JS searched the literature and wrote the manuscript. JP designed the figures. JP, GK, and JS edited and approved the final version of the manuscript.

### Conflict of interest statement

The authors declare that the research was conducted in the absence of any commercial or financial relationships that could be construed as a potential conflict of interest.
